# Elucidating the genotype–phenotype map by automatic enumeration and analysis of the phenotypic repertoire

**DOI:** 10.1038/npjsba.2015.3

**Published:** 2015-09-28

**Authors:** Jason G Lomnitz, Michael A Savageau

**Affiliations:** 1 Department of Biomedical Engineering, University of California, Davis, CA, USA; 2 Microbiology Graduate Group, University of California, Davis, CA, USA

## Abstract

**Background:**

The gap between genotype and phenotype is filled by complex biochemical systems most of which are poorly understood. Because these systems are complex, it is widely appreciated that quantitative understanding can only be achieved with the aid of mathematical models. However, formulating models and measuring or estimating their numerous rate constants and binding constants is daunting. Here we present a strategy for automating difficult aspects of the process.

**Methods:**

The strategy, based on a system design space methodology, is applied to a class of 16 designs for a synthetic gene oscillator that includes seven designs previously formulated on the basis of experimentally measured and estimated parameters.

**Results:**

Our strategy provides four important innovations by automating: (1) enumeration of the repertoire of qualitatively distinct phenotypes for a system; (2) generation of parameter values for any particular phenotype; (3) simultaneous realization of parameter values for several phenotypes to aid visualization of transitions from one phenotype to another, in critical cases from functional to dysfunctional; and (4) identification of ensembles of phenotypes whose expression can be phased to achieve a specific sequence of functions for rationally engineering synthetic constructs. Our strategy, applied to the 16 designs, reproduced previous results and identified two additional designs capable of sustained oscillations that were previously missed.

**Conclusions:**

Starting with a system’s relatively fixed aspects, its architectural features, our method enables automated analysis of nonlinear biochemical systems from a global perspective, without first specifying parameter values. The examples presented demonstrate the efficiency and power of this automated strategy.

## Introduction

Biological systems display an enormous variety of phenotypes that emerge through complex interactions between their genotype and environment. Relating genotype and environment to phenotype is difficult,^1^ and at a deep level requires mathematical models of the organism’s intervening biochemistry. Realizing an appropriate model for most biological systems is challenging because there are large number of parameters and their values are largely unknown and difficult to measure or estimate.^2,3^ The phenotypes manifested by the model representing the system are the result of several mappings: genome to genotypically determined structural parameters of the model, environment to environmentally determined input parameters of the model, and the gene-by-environment space of model parameters to the quantitative phenotypes of the model representing the biochemical system. In view of this last mapping, we have defined ‘phenotype’ as the attributes of a biochemical system in steady-state determined by a unique set of values in the gene-by-environment space of model parameters.

Elucidation of the mechanistic link from genotype and environment to phenotype is a nearly intractable problem for two primary reasons. (a) The phenotype corresponding to a unique point in parameter space is the manifestation of a complex system that is analytically intractable and requires sampling numerous simulations for its characterization. (b) The parameter space represents an infinite number of phenotypes in a homogenous continuum. Thus, a high-dimensional parameter space can only be sparsely sampled, and it is unlikely that the full repertoire of phenotypes latent in any particular system design will be revealed. Moreover, every complex model has hidden fragilities, which under certain combinations of environment and genotype manifest themselves in unintended and dysfunctional consequences, and it is a fundamental challenge to identify these.

In previous attempts to address this challenge we developed an approach that partitions parameter space into a finite number of ‘chunks’ or regions (technically, space-filling convex irregular polytopes). The partitioning is not arbitrarily imposed, but objectively determined by the system itself. We defined this space as the ‘system design space’, which has a finite number of discrete and structured regions, in contrast to parameter space, which is infinite, continuous and homogenous. The characteristic phenotype throughout a region is defined as a ‘qualitatively distinct phenotype’, and we simply refer to these as the phenotype of a region in design space when the context makes this clear. The collection of qualitatively distinct phenotypes (or phenotypic regions) is defined as the ‘phenotypic repertoire’ of the system. Moreover, each qualitatively distinct phenotype is characterized by a tractable subsystem model that allows efficient analysis of the phenotype and ranking of its relative fitness according to objective quantitative criteria. Thus, partitioning the gene-by-environment space of model parameters into the system design space largely avoids the sampling problem and, by efficiently identifying phenotypic regions of interest, it facilitates a focused analysis to refine the phenotype characterization using conventional techniques. A simple mass-action example, amenable to a completely analytical as well as intuitive treatment, can be found in a recent review.^4^

We previously used design space methodology to characterize several natural systems^5–9^ and a number of synthetic constructs.^10,11^ In each case, the approach started with experimentally determined parameter values for an established model. An early application of this approach to the oxygen stress response system in human erythrocytes revealed three qualitatively distinct phenotypes whose ranked fitness revealed a physiological, pathological and potentially lethal phenotype.^12^ Experimental data for 67 well-characterized variants of the G6PD enzyme, the key component of the stress response system, exhibited two of the three phenotypes: variants from ‘normal’ individuals were typically associated with the physiological phenotype and those from ‘hemolytic’ individuals were typically associated with the pathological phenotype. None of these 67 well-characterized variants was associated with the phenotype having the worst fitness characteristics, which we suggest might indicate that such variants are lethal. However, as systems become larger and more complex with relatively few known parameter values, systematic and automated strategies that identify, analyze and rank their qualitatively distinct phenotypes become essential.

Here we introduce a new strategy, based on system design space methodology, that inverts the previous order of analysis and automates the entire process. The strategy starts with the relatively fixed, architectural, features of a model—as distinct from its parameters (for more on this distinction see System Architecture in Supplementary Online Methods)—and proceeds automatically in four parts: (a) enumerating the phenotypic repertoire without specifying parameter values, (b) finding a set of parameter values for the realization and characterization of each qualitatively distinct phenotype, (c) identifying a two-dimensional slice of system design space that allows simultaneous visualization of several regions representing qualitatively distinct phenotypes and (d) identifying an ordered sequence of phenotypes capable of modeling specific functional characteristics of natural systems or guiding construction of synthetic systems to achieve desired functions. We demonstrate validity of the automated strategy, without specifying parameter values, by applying it to a previously analyzed gene circuit oscillator, which was based on experimentally measured and estimated parameter values. The demonstration is extended to a general class of two-gene circuits, showing that it not only reproduces earlier results but also reveals new results previously overlooked.

## Materials and Methods

Details in Supplementary Online Methods provide (a) background on the design space methodology, (b) a simple example (see Supplementary Figure M1) as a vehicle to introduce the more abstract and technical aspects for each part of the strategy and (c) a description of the methodology applied to a general class of two-gene circuits. The Results section provides in parallel a more intuitive description of the same strategy and the application to synthetic circuits for the design of an oscillator. Readers immediately interested in the technical aspects might wish to proceed directly to Supplementary Online Methods and then return to the new results presented here.

## Results

**Figure 1:**
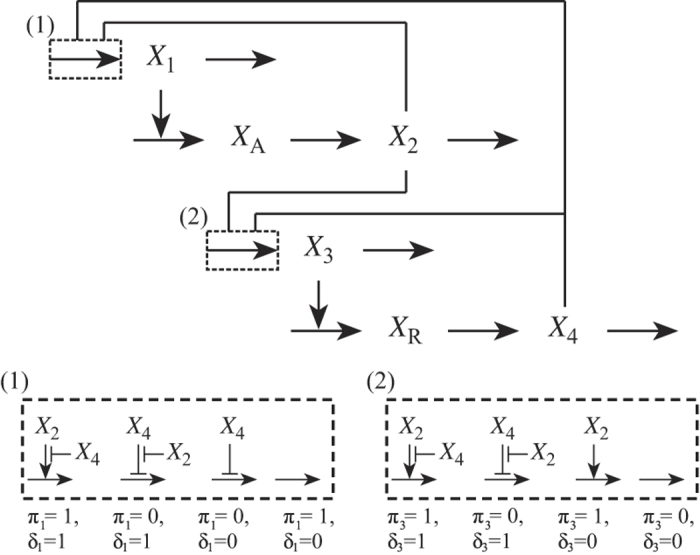
General class of two-gene circuits with one activator and one repressor. The species represent activator mRNA, *X*_1_; nascent activator protein, *X*_A_; mature activator protein, *X*_2_; repressor mRNA, *X*_3_; nascent repressor protein, *X*_R_; and mature repressor protein, *X*_4_. Barbed arrows represent stimulatory influences; blunt arrows represent inhibitory influences. Arrows ending on the shaft of other arrows represent influence on a given process; horizontal arrows represent mass flow. The alternative modes of transcription control are shown inside the large dashed boxes. The alternatives include two dual, one single and one constitutive mode of transcription control. The *π* and *δ* are binary indices that define the mode of transcriptional control. The primary mode of transcriptional control involves an activator (*π*=1) or a repressor (*π*=0). The transcriptional control involves dual (*δ*=1) or single (*δ*=0) regulators. The combination *δ*_1_=0 and *π*_1_=1 (or *δ*_3_=0 and *π*_3_=0) indicates a constitutive mode of transcription control for the activator (or repressor). For example, the relaxation oscillator design is represented by *π*_1_=1, *δ*_1_=1, *π*_3_=1 and *δ*_3_=0. Note: the single modes of transcriptional control of the activator (Box 1) and repressor (Box 2) are different.

We first illustrate our strategy by reanalyzing a two-gene relaxation oscillator circuit that displays rich behaviors including hysteresis and oscillations.^10^ Then, we perform an automated analysis for the class of two-gene circuitry involving an activator and a repressor as shown in Figure 1.

### Two-gene relaxation oscillator

We apply our strategy to a two-gene synthetic oscillator that has been shown to exhibit damped oscillations.^13^ Its design is similar to that in Figure 1 with architectural indices given by *π*_1_=1, *δ*_1_=1, *π*_3_=1, and *δ*_3_=0. We previously formulated a mechanistic model, incorporated experimentally estimated parameter values, performed conventional bifurcation analysis as well as our design space analysis, and showed that the design is capable of exhibiting sustained oscillations.^10^ Our goal here is to test the extent to which our automated methods reproduce the previous results, but without experimentally estimated parameter values.

**Figure 2:**
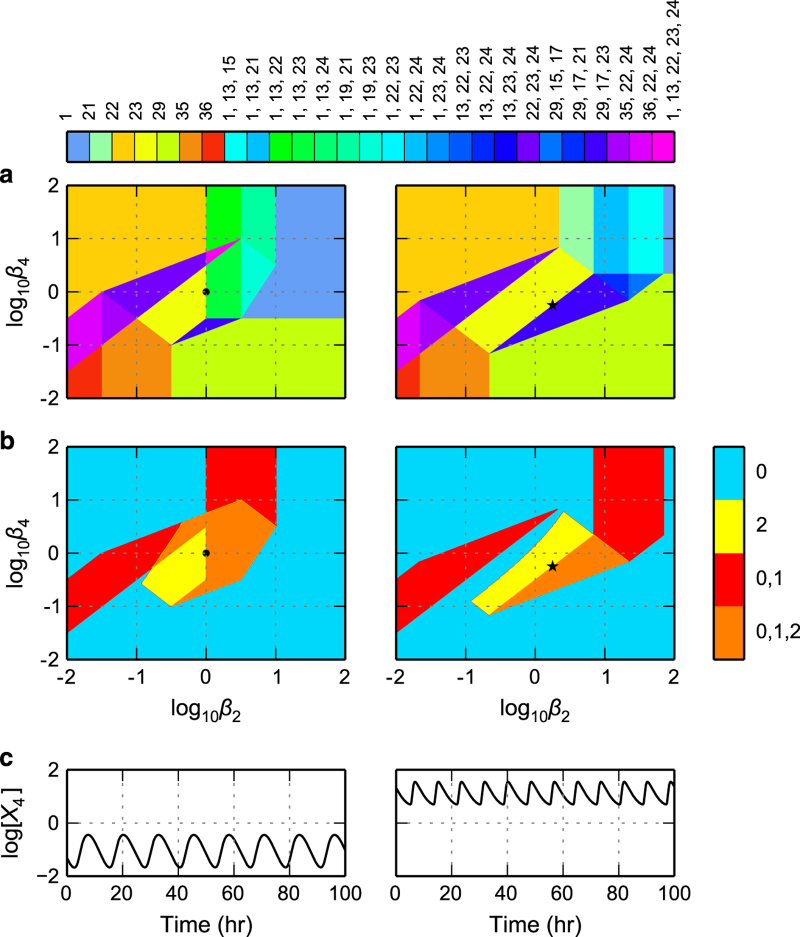
Analysis of the relaxation oscillator design centered on the set of parameter values automatically determined for the oscillatory phenotype. Results from the automated strategy without specifying values for the parameters (left panels) are compared with results from a previous study^10^ based on experimentally measured and estimated values for the parameters (right panels). (**a**,**b**) System design space with the effective rate constant for inactivation of the two regulators on the *x* and *y* axes. (**a**) Enumeration of the qualitatively distinct phenotypes identified by color. (**b**) The number of eigenvalues with positive real part represented as a heat map on the *z* axis: blue for 0 eigenvalues with positive real part (mono-stability); red for an overlap of Cases consisting of one with 1 and two with 0 eigenvalues having positive real part (bi-stability); yellow for two complex conjugate eigenvalues with positive real part (unstable focus); orange for an overlap of Cases consisting of one with 0, one with 1 and one with 2 eigenvalues having positive real part. The overlaps represented by orange regions correspond to three fixed points: a stable node, an unstable node and an unstable focus; boundaries between orange and yellow regions have the potential for Saddle-Node into Limit Cycle (SNIC) bifurcations that produce transitions between stable steady-state behavior and large-amplitude oscillations.^10^ (**c**) Temporal behavior of repressor concentration *X*_4_ determined by simulation of the full system with parameter values from the automatic strategy (● in left panels) and with experimentally determined values from the previous study (★ in right panels). Note that the values of the parameters on the *x* and *y* axes of both panels are near the center of the region of potential oscillation (yellow+orange). The values in the left panel are automatically determined, whereas those in the right panel are manually selected to be near the center of the region of potential oscillation.

The first part of our automated strategy involves enumerating the qualitatively distinct phenotypes of the system to identify its complete phenotypic repertoire (e.g., see Supplementary Table M1). The mechanistic model and the meaning of its parameters can be found in Supplementary Online Methods. It has a maximum of 36 potentially valid qualitatively distinct phenotypes, as defined within the framework of the design space approach.^14^ However, our automatic enumeration reveals that only 15 of these are valid somewhere in parameter space. The phenotypic repertoire of the system is listed in Table 1 and shown graphically in the left panel of Figure 2a by an arbitrary color-coded Case no. in design space.

The second part of our automated strategy involves finding a set of parameter values that realizes each qualitatively distinct phenotype and facilitates their further characterization (e.g., see Supplementary Figure M2). The steady-state solution, or fixed point, of the S-system model identified with each phenotype can be determined analytically, and diverse steady-state and local dynamic characteristics can then be determined.^15,16^ As shown in the last column of Table 1, we find phenotypes that are stable, exponentially unstable and oscillatory unstable. The number of eigenvalues with positive real part is the phenotypic characteristic plotted as a heat map in the left panel of Figure 2b. The case with two complex conjugate eigenvalues having positive real part is consistent with limit cycle oscillations arising through Hopf bifurcations.^10,11^ Thus, the results of our stability analysis show that only one phenotype, Case 23, has the potential to exhibit sustained oscillations. By using the automated strategy with a parameter-independent approach we have obtained the same oscillatory phenotype as previously obtained with estimated and experimentally measured parameter values,^10^ as well as similar neighboring phenotypes in design space (reproduced in the right panels of Figure 2 for comparison).

The third part of our automated strategy involves identification of parameter values that allow different phenotypes to be located in the same view (slice) of design space (e.g., see Supplementary Figure M3). A special case of this capability allows us to find the maximum number of qualitatively distinct phenotypes capable of being visualized together in a plane (e.g., see Supplementary Figure M4), 11 as shown for this example in Figure 2a: Cases 1, 13, 17, 19, 21, 22, 23, 24, 29, 35 and 36. Thus, our automated design space strategy has (a) efficiently reproduced previous results for this relaxation oscillator by identifying the phenotype that exhibits the potential for oscillatory behavior and by obtaining a concrete example showing sustained oscillations, and (b) demonstrated that the design space in the left panel of Figure 2a contains most of the phenotypic repertoire and thus is representative of the rich behavior this system is capable of exhibiting.

### General class of two-gene oscillator designs

We previously formulated mechanistic models for seven designs, involving one of three architectures and four modes of transcriptional control, and compared them under conditions that maximize their potential for sustained oscillation.^11^ Here we use the same three parts of the strategy described in the previous subsection to analyze the broader class of 16 two-gene circuits shown in Figure 1 with four modes of transcriptional control for both regulators. The mathematical model for this general class and the detailed methods of analysis are included in Supplementary Online Methods.

For each design we apply our automated strategy to (a) enumerate the qualitatively distinct phenotypes, (b) obtain a set of parameter values for each of the valid phenotypes and determine the number of potential oscillatory phenotypes, and (c) determine the maximum number of oscillatory phenotypes that can be realized and visualized together in a plane. The results from the first two parts are summarized in Table 2, where the strategy identified seven of the 16 designs that are unable to produce oscillatory phenotypes. These designs lack the essential delay and co-operativity required for an unstable focus and a stable limit-cycle oscillation to exist. For example, the D.7 design (with indices *π*_1_=1, *δ*_1_=0, *π*_3_=0, and *δ*_3_=1 in Figure 1) has constitutive expression of activator and a negative auto-regulatory feedback loop, which is antagonized by the activator (i.e., a repressor-primary mode of transcriptional control^11^). This is a simple three-step pathway with an effective co-operativity of two. It is known that a system with three steps needs an effective co-operativity more than eight.^17,18^ Thus, these seven designs are not expected to exhibit sustained oscillations.

Beyond these expected cases, the automated analysis revealed surprising new results. We found two additional designs within the general class of 16 that can exhibit oscillatory behavior (D.11 and D.3) and cases of multiple oscillatory phenotypes for a given design. The first of the newly identified designs (D.11: *π*_1_=0, *δ*_1_=0, *π*_3_=1, *δ*_3_=1) has the potential for oscillation with two different phenotypes: the first phenotype has interactions that are effectively equivalent to those of a negative-only architecture (D.9: *π*_1_=0, *δ*_1_=0, *π*_3_=1, *δ*_3_=0); the second phenotype has interactions that cannot be reduced to those found in one of the designs we previously identified. The second of the newly identified designs (D.3: *π*_1_=0, *δ*_1_=0, *π*_3_=0, *δ*_3_=1) has the potential for oscillation in one phenotype with effective interactions that also cannot be reduced to one of the original designs.^11^ The new designs, D.11 and D.3, involve repressor-only control of activator transcription and dual modes of control for repressor transcription. These designs share the same general architecture but involve alternative dual modes of control of repressor transcription; D.11 is the activator-primary design (positive feedback antagonized by repressor) and D.3 is the repressor-primary design (negative feedback antagonized by activator). Hence, through our automatic strategy we have identified two entirely new designs that bring the total to nine irreducible designs within this general class that have the potential for oscillatory behavior.

In Table 2 we show that our automated strategy identified five of these nine designs with multiple phenotypes that exhibit the potential for oscillation. These additional oscillatory phenotypes result from interactions that are equivalent to those found in one of the other nine irreducible oscillatory designs. Amongst all the designs, D.12 has the largest number of oscillatory phenotypes, with a total of four represented by Cases 16, 18, 43 and 45. Case 45 maximizes the interactions within the system and has the architecture characteristic of this oscillator. Cases 16 and 43 have dominant terms that reduce the system to one of the original designs (a simple negative-only feedback loop, D.9, and a relaxation oscillator, D.10, respectively). Case 18 has dominant terms that reduce the system to one of the new oscillator designs we have identified in this paper (D.3).

**Figure 3:**
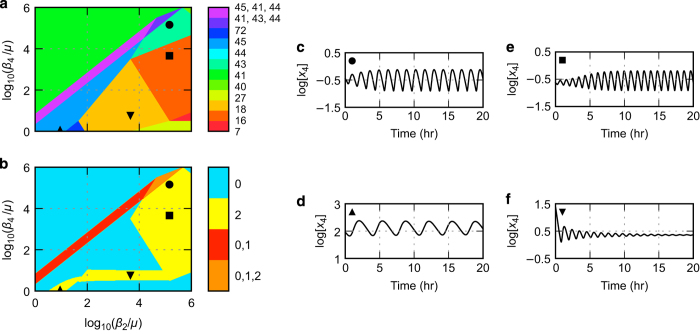
Example of an ensemble of four oscillatory phenotypes in a two-dimensional slice of system design space for the D.12 design. (**a**,**b**) System design space with the effective rate constant for inactivation of the two regulators on the *x* and *y* axes, normalized with respect to the growth rate, *μ*, with a 1 h doubling time. See caption of Figure 2 for details. (**c**–**f**) Temporal behavior of normalized repressor concentration *x*_4_ determined by simulation of the full system within the phenotypic regions of potentially oscillation in panels (**a**,**b**) indicated by the symbols in the upper-left corners (regions 43 ●, 16 ■, 45 ▲ and 18 ▼). It should be noted that sustained oscillations may dynamically cycle through different qualitatively distinct phenotypes in state space.^11^

In the third part of our strategy, we pose the question: is there a set of parameter values such that all four of the oscillatory phenotypes of design D.12 can be simultaneously realized within the same two-dimensional slice of design space (i.e., where all but two parameters are fixed)? The task of identifying parameter values that simultaneously realize multiple observed or desired phenotypes has important implications for critical hypothesis testing; yet, this task is extremely challenging using conventional methodologies. By applying our strategy we readily identify a slice in which all four potential oscillatory phenotypes are simultaneously visualized within adjacent regions, as shown in Figure 3a.

The actual limit-cycle oscillations have parameter values that reside within a single contiguous area of design space that includes parts of all four phenotypic regions (Figure 3b). Simulations of the full system using parameter values located within each of these four regions shows three examples of sustained oscillation and one example of damped oscillation. Thus, our results have automatically determined that the ensemble of four distinct oscillator phenotypes for the D.12 design can be simultaneously realized within the same two-dimensional slice of design space. This approach to the identification of ensembles of phenotypes also can be used in the fourth part of our four-part strategy to automatically order ensembles of phenotypes to achieve a specific sequence of functions to replicate natural systems or to rationally engineer synthetic constructs. The details of this innovation and a specific example can be found in Supplementary Online Methods.

## Discussion

The integration of rigorously defined phenotypes into a system design space allows the qualitatively distinct phenotypes of a system to be identified, enumerated, characterized and compared. The landmarks in system design space represent particular constellations of parameters that define relevant design principles.^7^ All these aspects of the design space strategy would be further enhanced if the strategy could be more fully automated. This has been the motivation for the work presented here. The automated strategy provides a novel method of model reduction with the advantage of efficiently identifying regions of interest in the overall phenotypic landscape. The behavior in these regions can then be examined with conventional methods to obtain a more refined analysis of the full system, particularly near boundaries between phenotypes where the differences tend to break down.^10^ Parameter values can be bounded and even fixed by specific constraints, and once constrained, the analysis can guarantee that parameters meet realistic requirements. Although scalability is an issue being actively explored, there is compensation in that computations can be performed in parallel (see Part 1 in Supplementary Online Methods).

As illustrations of these innovations we have provided applications to different gene circuit designs exhibiting rich dynamic behaviors that include bi-stability and limit-cycle oscillations. In the application to a general class of two-gene circuits we identified nine out of 16 designs capable of exhibiting sustained oscillatory behavior, with two being new designs overlooked in an earlier study. Moreover, for many designs in this class we identified multiple phenotypes capable of exhibiting oscillatory behavior. For one design we found an ensemble of four distinct oscillatory phenotypes that can be visualized within a single relevant slice of design space.

This identification and characterization of the phenotypic potential of nonlinear models can serve as a rigorous basis for model discrimination in the process of hypothesis testing. Once a working hypothesis has been formulated in terms of system architecture, the task of extracting and testing its latent implications becomes a challenge because of the large number of unknown and in many cases unknowable parameter values. The automated design space strategy introduced in this paper offers four specific tools that address this challenge. First is an enumeration of the phenotypic repertoire for a system that does not require values for its parameters. Second is the generation of a specific set of parameter values for the realization of each qualitatively distinct phenotype. Third is the discovery of ensembles of phenotypes that can be simultaneously realized in particular slices of design space to improve visualization and understanding of transitions between phenotypic regimes. Fourth is the ability to identify ensembles of phenotypes that can be ordered to achieve a specific sequence of desirable behaviors (e.g., see Supplementary Figure M5 and Part 4 in Supplementary Online Methods). Automating these steps in the design space strategy allows for faster cycles of hypothesis testing.

The automated design space strategy is focused on nonlinear systems governed by chemical and biochemical kinetics. Since these characterize the vast majority of cellular processes, this strategy is likely to be broadly applicable in biology for understanding natural systems and constructing synthetic systems. We speculate that other types of nonlinearity in other types of systems also might be amenable to this strategy because a wide variety of nonlinear systems can be recast into the generalized mass action system of equations that is at the heart of the design space approach.

## Figures and Tables

**Table 1:** Enumeration of the phenotypic repertoire and potential dynamic behaviors for the relaxation oscillator design

*Case no.*	*Case signature*	*No. of eigenvalues with positive real part*
1	111111111111	0
7	111121111111	0
8	111121111121	0
13	211111111111	1
15	211111112111	0
17	211111113111	1
19	211121111111	1
20	211121111121	1
21	211121112111	0
22	211121112121	0
23	211121113111	2
24	211121113121	1
29	311111113111	0
35	311121113111	0
36	311121113121	0

Each design has a unique System Signature defined by a pair of integers for each equation of the system; the first of each pair indicating the number of positive terms and the second the number of negative terms in each equation. The System Signature in this application is [311121113121]. Each potential phenotype has a Case Signature, analogous to the System Signature, with the first of each pair signifying a particular term among the positive terms and the second a particular term among the negative terms in each equation. The potential phenotypes are given arbitrary sequential Case Numbers according to conventional digital counting of their Signatures. In this application: Case 1, (111111111111); Case 2, (111111111121); Case 3, (111111112111); Case 4, (111111112121); Case 5, (111111113111); …; Case 36, (311121113121). Note that 21 of the 36 potential phenotypes are not realizable; e.g., Cases 2 through 6. The number of eigenvalues with positive real part indicates whether the phenotype is stable with zero, exponentially unstable with one, or oscillatory unstable with two that are complex conjugate. Eigenvalues are determined using the set of parameters automatically determined for each of the phenotypes. For further details see Supplementary Online Methods.

**Table 2:** Summary of global properties for the 16 designs in the general class of two-gene circuits

*Design identifier*	*Indices for the mode of control*^a^	*Phenotypic fraction*^b^	*No. of oscillatory phenotypes*
D.1	*π*_1_=0, *δ*_1_=0, *π*_3_=0, *δ*_3_=0	6/16	0
D.2	*π*_1_=0, *δ*_1_=1, *π*_3_=0, *δ*_3_=0	10/36	0
D.3	*π*_1_=0, *δ*_1_=0, *π*_3_=0, *δ*_3_=1	15/36	1
D.4	*π*_1_=0, *δ*_1_=1, *π*_3_=0, *δ*_3_=1	25/81	2
D.5	*π*_1_=1, *δ*_1_=0, *π*_3_=0, *δ*_3_=0	4/16	0
D.6	*π*_1_=1, *δ*_1_=1, *π*_3_=0, *δ*_3_=0	10/36	0
D.7	*π*_1_=1, *δ*_1_=0, *π*_3_=0, *δ*_3_=1	10/36	0
D.8	*π*_1_=1, *δ*_1_=1, *π*_3_=0, *δ*_3_=1	25/81	1
D.9	*π*_1_=0, *δ*_1_=0, *π*_3_=1, *δ*_3_=0	9/16	1
D.10	*π*_1_=0, *δ*_1_=1, *π*_3_=1, *δ*_3_=0	15/36	2
D.11	*π*_1_=0, *δ*_1_=0, *π*_3_=1, *δ*_3_=1	15/36	2
D.12	*π*_1_=0, *δ*_1_=1, *π*_3_=1, *δ*_3_=1	25/81	4
D.13	*π*_1_=1, *δ*_1_=0, *π*_3_=1, *δ*_3_=0	6/16	0
D.14	*π*_1_=1, *δ*_1_=1, *π*_3_=1, *δ*_3_=0	15/36	1
D.15	*π*_1_=1, *δ*_1_=0, *π*_3_=1, *δ*_3_=1	10/36	0
D.16	*π*_1_=1, *δ*_1_=1, *π*_3_=1, *δ*_3_=1	25/81	2

a The meaning of the *π* and *δ* symbols is described in the caption of Figure 1.

b The phenotypic fraction is shown as the number of valid phenotypes divided by the maximum number of potential phenotypes.
