# Supplementary material for: Elucidating the genotype–phenotype map by automatic enumeration and analysis of the phenotypic repertoire
Source: NPJ Syst Biol Appl. 2015 Sep 28;1:15003–. doi: 10.1038/npjsba.2015.3 (PMC4794114; doi:10.1038/npjsba.2015.3)
Supplement: Supplementary Online Methods [file npjsba20153-s1.doc]

**ONLINE METHODS**

**Elucidating the Genotype-Phenotype Map by Automatic Enumeration and Analysis of the Phenotypic Repertoire**

**Jason G. Lomnitz^1^ and Michael A. Savageau^1,2,*^.**

**^1^**Department of Biomedical Engineering, and **^2^**Microbiology Graduate Group, University of California, Davis, CA 95616 USA

* To whom correspondence should be addressed. Tel: +01 530 754 7350; Fax: +01 530 754 5739; Email: [masavageau@ucdavis.edu](mailto:masavageau@ucdavis.edu)

**INTRODUCTION**

We first give a brief description of three preliminaries. The methods underlying the innovations achieved by our automated strategy are then described in detail by using the vehicle of a simple model to facilitate the four-part presentation: (1) enumeration of the repertoire of qualitatively distinct phenotypes for a system; (2) generation of parameter values for any particular phenotype; (3) simultaneous realization of values for different phenotypes in parameter space to visualize transitions from one phenotype to another, in critical cases from functional to dysfunctional behavior; and (4) identification of ensembles of phenotypes whose expression can be phased to achieve a specific sequence of functions for rationally engineering synthetic constructs. Finally, the details of these methods are presented for an application to the automatic identification of the phenotypic repertoire for a class of genetic oscillator designs. The corresponding results are described and discussed in the main text.

**SYSTEM DESIGN SPACE PRELIMINARIES**

In this section, we describe three aspects at the system design space methodology that are at the foundation of our automated strategy for providing a global perspective on the system’s phenotypic repertoire. First is the system architecture for a mechanistic model consisting of nonlinear ordinary differential equations, second is the recasting of these equations into a standard nonlinear form, and third are generic definitions of systemic phenotypes.

***System Architecture***

By system architecture we mean those features that remain fixed independent of the parameter values that characterize particular instantiations of a system design. Important architectural features include (a) the network topology of interactions, (b) the signs of the interactions, and (c) the number of binding sites involved in the interactions. The first two can be estimated by using current high-throughput technologies and methods for identifying causal connectivity^1,2^; similarly, the number of binding sites is routinely determined in the case of specific motifs in the DNA sequence and the number of subunits in multimeric proteins.

The number of binding sites in turn manifests itself in the kinetic orders and Hill numbers of the rate laws that describe the flow of mass and information within the system. In these cases, the kinetic orders typically have small integer values corresponding to the maximum number of binding sites in the mechanism^3^. Even for complex mechanism where the number of binding sites is unknown and the kinetic orders must be approximated, sampling a small number of alternatives may be sufficient since the range of empirical kinetic orders for the vast majority of mechanisms is relatively small, typical between 0 and 4. Once approximate kinetic orders are measured, estimated, or assumed, they become part of the model architecture and are no longer considered parameters from the perspective of our strategy.

These architectural features are fixed for a given system design. The rate constant and binding parameters that specify any particular system are typically unknown and difficult to measure or estimate. There are no high-throughput technologies on the horizon to measure these kinetic parameters *in situ*. Thus, it is critically important to determine the system architecture and obtain a global perspective on the phenotypic repertoire before launching a time-consuming and expensive effort to determine numerical values for the parameters of a model.

To facilitate the presentation, the automated strategy is first illustrated with the simplified conceptual model shown in Figure M1. This model is then converted into a mathematical model that involves ordinary differential equations. The rate of synthesis of *X*_1_ is described by a rational function with a basal rate of synthesis at low concentrations of *X*_2_, an increase in the rate of synthesis within the regulatable regime as the concentration of *X*_2_ increases, and a maximum rate of synthesis at high concentrations of *X*_2_. The rate of synthesis of *X*_2_ is described by a similar rational function that is dependent on the concentrations of both *X*_1_ and *X*_3_. This rate has a linear response to the concentration of *X*_1_, whereas it has a sigmoidal response to the concentration of *X*_3_. The rate of loss of both *X*_1_ and *X*_2_ is assumed to be a first-order process such as dilution due to exponential growth. The equations that represent this system are the following

[1]

[2]

where the dimensionless kinetic orders for *X*_1_ activation by *X*_2_ and *X*_2_ activation by *X*_3_ are assumed to be 2, the *K_i_* parameters represent the concentration of activator for half-maximal activation, the *β_i_* parameters represent first-order rate constants for loss in units of inverse time, the *α_i_* parameters represent the basal rate of transcription for the *i*-th species, with appropriate units for dimensional consistency, and the maximum rate is *α_i_*⋅*ρ_i_*. The dimensionless parameters representing the capacity for activation are *ρ_i_* > 1; this ensures that the signs of the interactions remain unchanged, which is part of the system architecture. This approach to converting a conceptual model into a mathematical model is applicable to a large class of biochemical systems.

***Recasting Equations into a Generic Form***

In order to apply the design space methodology, we must recast the model into a generic form, and this can be done exactly through a defined series of steps^4^. Here, the generic form, a generalized mass action (GMA) system of equations, is obtained simply by defining new auxiliary variables for the denominators of Eqs. 1 and 2, and expanding the numerators. The result is a set of differential equations plus a set of algebraic constraints,

[3]

[4]

[5]

[6]

In the system design space methodology, system parameters (rate constants, regulatory capacities, and effective K_M_ values) and independent variables (only *X*_3_ in this example) are treated equally and thus we refer to both as parameters of the model.

*Grouping terms*

Starting with a system of *M* equations in the GMA form, where the right-hand side of each equation is a sum of *terms* consisting of products of power laws. Each term has either a positive or negative coefficient, and the terms of an equation are grouped into *positive terms* and *negative terms*.

*System signature*

The *system signature* is defined as a list given by the number of positive and negative terms in each equation: [*P*_1_, *N*_1_, *P*_2_, *N*_2_, …, *P_M_*, *N_M_*], where *P_i_* and *N_i_* represent the number of positive and negative terms in the *i*-th equation, respectively. The system signature can be represented in a more compact form by removing the commas and spaces to yield [*P*_1_*N*_1_*P*_2_*N*_2_…*P_M_N_M_*], and values with multiple digits are represented within parentheses. The system defined by Eqs. 3-6 has 2 positive terms and 1 negative term in each of the equations. Thus, *P_i_* = 2 and *N_i_* = 1 for *i =* {1, 2, 3, 4} and yields the system signature [21212121].

*Dominant terms*

At any point in time, each term of the system has a particular magnitude determined by the values of the variables and parameters of the system. For each equation, we identify the largest positive term, which we call the *dominant positive term*, and the largest negative term, which we call the *dominant negative term.* Therefore, a *dominant term* is defined as the largest term of a given sign for an equation in the GMA form. For example, if we assume *X_4_* = 1, *K*_2_ = 10, *X*_2_ = 0.1, *ρ*_1_ = 100 and *α*_1_ = 1 and we substitute these values into Eq. 3, we find that the first and second positive terms are equal to 1 and 0.01, respectively. The first term is larger than the second and therefore it is the dominant positive term for Eq. 3. Because there is only one negative term in Eq. 3, this too is considered a dominant term.

*Dominant sub-systems*

We define a *dominant sub-system* of the intact system where only the dominant terms are retained, and all other terms are neglected and removed from the system equations. The result is a nonlinear sub-system or *S-System* with one positive term and one negative term.

For example, assume that the first positive and the first negative terms are dominant in Eqs. 3-5, and the second positive and the first negative terms are dominant in Eq. 6. If we retain the dominant terms, and neglect all non-dominant terms, we obtain the following dominant S-system:

[7]

[8]

[9]

[10]

*Case signatures and case numbers*

Every S-system can be uniquely identified by its *case signature* and *case number*. The *case signature* is the sequence of numbers indicating the index of the dominant positive and dominant negative term in each equation: (*p*_1_, *n*_1_, *p*_2_, *n*_2_, …, *p_M_*, *n_M_*), where *p_i_* and *n_i_* represent the index of the dominant positive and dominant negative term for the *i*-th equation, respectively. Like the system signature, the case signature can be represented in a more compact form by removing the commas and spaces, (*p*_1_*n*_1_*p*_2_*n*_2_…*p_M_n_M_*). *Case numbers* are uniquely associated with the sequence of case signatures, from the smallest (111111…11) to the largest (*P*_1_*N*_1_*P*_2_*N*_2_…*P_M_N_M_*).

For example, the intact system in Eqs. 3-6 with system signature [212121] has 16 S-systems. These can be listed according to their case number and case signature as follows,

Case 1 with signature (11111111)

Case 2 with signature (11111121)

Case 3 with signature (11112111)

…

Case 16 with signature (21212121)

The dominant S-system defined by Eqs. 7-10 has dominant terms with the following indices, *p_i_* = 1 and *n_i_* = 1 for *i* = {1, 2, 3} and *p*_4_ = 2 and *n*_4_ = 1. The indices are listed to identify the corresponding case signature (11111121) and the associated case number Case 2.

*Dominance conditions*

Not all dominant S-systems are valid. The validity of a dominant S-system depends on a set of *dominance conditions* that must be satisfied. The dominance conditions are the inequalities that arise from assuming that one term, the dominant term, is larger than every other term of the same sign in a given equation. For example, the dominant S-system given by Eqs. 7-10 involves a particular combination of dominant terms. For the dominant S-system to be valid, every dominant term must be larger than the remaining terms of the same sign in each equation. This is expressed mathematically by the following dominance conditions,

[11]

[12]

[13]

[14]

In addition, any architectural constraints are added to the dominance conditions because they too must be satisfied. Thus, the constraints ensuring that the regulators are activators are added as well,

[15]

[16]

*Boundary conditions in system design space*

Substituting the steady-state solution of a dominant S-system into the dominance conditions for its validity gives rise to a set of inequalities involving all the parameters and only the parameters of the full system. For example, the dominant S-system given by Eqs. 7-10, at steady state, can be transformed into logarithmic coordinates to yield a set of linear equations that can be readily solved,

[17]

[18]

[19]

[20]

The boundary conditions are obtained by substituting the steady-state solution in Eqs. 17-20 into the dominance conditions in Eqs. 11-16, which yields the following linear inequalities in logarithmic coordinates,

[21]

[22]

[23]

[24]

[25]

These boundary conditions must be satisfied for the potential S-system to be valid. Inspection of Eqs. 22, 23 and 25 reveals an inconsistency among the boundary conditions and therefore this S-system is *invalid*.

In general, the steady state is a solution to a system of linear equations in the logarithms of the variables and parameters, and the dominance conditions are likewise a system of linear inequalities in the logarithms of the variables and parameters. Thus, the boundary conditions can be expressed in the following matrix form^5^:

[26]

*U_i_* is a *N × p* matrix of architecturally-fixed constants for the *i*-th case, where *p* is the number of parameters of the system and *N* is the number of boundary conditions; *z_i_* is a column vector of *N* numerical values representing the logarithms of stoichiometric constants (in this simple example the values are all 0) for the *i*-th case; and *y* is a column vector of *p* values representing the logarithm of the system parameters.

The boundary conditions for all the valid S-systems partition parameter space into a finite number of “chunks” or regions (technically, space-filling convex irregular polytopes). The partitioning is not arbitrarily imposed, but objectively determined by the system itself. We defined this space as the *system design space*, which has a finite number of discrete and structured regions, in contrast to parameter space, which is infinite, continuous and homogenous. In this general approach the dependent variables of the system are eliminated and all the landmarks in design space are represented explicitly by particular constellations of the parameters of the original system.

These concepts form the basis for our generic definition of systemic phenotypes, both quantitative and qualitative, and the characterization of the phenotypic repertoire in system design space^5,6^.

***Generic Definition of Systemic Phenotypes***

*Quantitative phenotype*

Any state of the GMA system that is a fixed point, or steady state of the system, we define as a *quantitative phenotype*. These phenotypes exhibit a variety of *phenotypic properties*, such as values for concentrations, fluxes, robustness, stability, and response times for small variations. Each quantitative phenotype has a corresponding set of dominant terms functioning within the intact system. It remains a fundamental challenge to find every fixed point for a system of nonlinear equations, more so for every combination of parameters and independent variables. The fact that there is an infinite number of quantitative phenotypes and that only a small sample of them can be characterized for any complex nonlinear system seems hardly any better than the vague concept of phenotype conveyed by ad hoc descriptive terms. Nevertheless, the infinite number of quantitative phenotypes have only a finite number of dominant S-systems, which suggests a natural grouping of phenotypes according to their dominant S-systems.

*Qualitative phenotypes*

The deconstruction of complex intractable biochemical systems into a finite number of tractable S-systems, and their visualization as a partitioning of parameter space into the system design space as described above, leads to boundaries mathematically defined by the system itself. In this general approach the dependent variables of the system are eliminated but all the parameters of the original systems are retained explicitly in the landmarks of design space (coordinates of all the vertices) as particular constellations of the genotypically determined parameters and the environmentally determined variables of the system.

A *potentially valid qualitatively-distinct phenotype* is defined by having a unique dominant S-system with a particular combination of dominant terms. However, not all combinations yield a valid phenotype: the nonlinear S-Systems that represent the qualitatively-distinct phenotypes may not have a steady-state solution, alternatively the boundary conditions that determine the region of validity may not be satisfied anywhere in design space. The number of potentially valid qualitatively-distinct phenotypes for a given system is the finite number of combinations of dominant terms, and the number of combinations of dominant terms is given by the product of numbers in the system signature, i.e. . For example, the system defined by Eqs. 3-6, with signature [21212121], has a maximum of 2⋅1⋅2⋅1⋅2⋅1⋅2⋅1 = 16 potentially valid qualitatively-distinct phenotypes.

A *qualitatively-distinct phenotype* is defined as a *potentially valid* one that has passed the test for validity described in the previous section. The system design space methodology uses this definition of a qualitative-distinct phenotype to deconstruct complex systems into nonlinear S-systems with mathematically defined boundaries within which the phenotypes are valid^5–7^. The boundaries define *phenotypic regions* that are convex polytopes in logarithmic coordinates. Phenotypic regions of different qualitatively-distinct phenotypes may overlap in design space, which corresponds to a single set of parameter values for which there is more than a single fixed point. Validity of overlapping regions can be determined in the same way as qualitatively-distinct phenotypes. Furthermore, they can be treated in the same manner as phenotypic regions in the context of our four-part automated strategy presented in the next section.

*Phenotypic repertoire*

The *Phenotypic repertoire* is defined as the collection of qualitatively-distinct phenotypes integrated into the space-filling structure called the *system design space*^6,7^.

**AUTOMATED FOUR-PART STRATEGY**

Our automatic strategy builds on the Design Space Toolbox 1.0 for Matlab®^5^, uses an alternative parallel implementation in open-source software, and incorporates new tools (currently under development) that automatically perform several additional steps in the analysis of a biochemical system. Our presentation of the methods starts with the basic architecture of a model that has been established, although none of the values for the parameters are known, and follows a four-part automatic strategy: (1) enumeration of the repertoire of qualitatively distinct phenotypes of a system; (2) generation of parameter values for any particular phenotype of the system; (3) co-localization of phenotypes in system design space to facilitate the visualization of phenotypic transitions, in critical cases from functional to dysfunctional behavior; and (4) identification of ensembles of phenotypes that can be ordered to achieve a specific sequence of functions for the rational engineering of synthetic constructs.

***Part 1: Enumeration Of Qualitatively-Distinct Phenotypes***

This first of our automated four-part strategy is made more concrete in the example defined by Eqs. 3-6. Recall from the previous section that this example has the following system signature [21212121] and a maximum of 16 potentially valid qualitatively-distinct phenotypes, or Cases. The complete set of case numbers and case signatures are shown in the first two columns of Table M1. The set of (valid) qualitatively-distinct phenotypes can be enumerated automatically without sampling parameter values using systematic methodologies. This is done for each case by (a) determining that a steady-state solution exists (through linear analysis in logarithmic coordinates^8^) and (b) determining if the boundary conditions define a feasible region in design space. The boundary conditions are linear inequalities in logarithmic space^6^ and are used to define a linear programming problem^5^. The task of determining if a feasible region exists somewhere in design space can be performed efficiently for a large number of variables with a large number of conditions^9^.

The current implementation of our method considers all combinations of potentially dominant terms and this gives a bound on the number of cases. However, it is a rather poor bound, since the rigorously defined valid cases are typically many fewer than the theoretical bound. Our method is considered ‘embarisingly paralizable’, since the cases can be independently analyzed in parallel. With our current parallel version, we have analyzed systems involving millions of case in minutes with twelve processors. Clearly, a large cluster will allow still larger systems to be examined. Furthermore, there are possibilities to prune the tree of possibilities, and we hope that in future this will give a more realistic bound on the number of cases that need to be examined. In any case, the currently automated features have significantly enhanced the capabilities of the design space methodology.

The results of our automatic enumeration for the simplified example are shown in the third column of Table M1, where only 9 of the 16 cases are valid somewhere in design space. This complete set of qualitatively-distinct phenotypes defines the phenotypic repertoire of the system.

***Part 2: Identification of Phenotype-Specific Sets of Parameter Values***

Given that the complete phenotypic repertoire of a system has been enumerated, how does one obtain meaningful sets of parameter values for a given phenotype? A naive approach is to sample parameter space until the desired phenotype is found. However, there are severe limitations to this approach: the number and nature of the behaviors is highly dependent on the density of sampling. If we were to look for qualitatively-distinct phenotypes by sampling 4 values for each of the 9 parameters in our simplified model, we would need to sample 4^9^ = 262144 values. Even with such a large number of samples, there is no guarantee that all the phenotypes would have been sampled, and the likelihood of missing behaviors altogether would be high. Our strategy offers a different approach to finding parameter values.

By virtue of the linear boundaries in logarithmic coordinates, our design space tools provide a powerful means to obtain a unique set of parameter values within the feasible region of any qualitatively-distinct phenotype. We achieve this by defining a linear programming problem with an arbitrary linear objective function. According to the fundamental theorem of linear programming^9,10^, if a solution exists it will be a basic feasible solution at an extreme point of the feasible region. Therefore, any solution to the linear programming problem yields a set of parameter values at a vertex of the phenotypic region in design space.

*Identifying interior phenotype-specific sets of parameter values*

It is more useful to acquire parameters values that are within the interior of a feasible region, where the behavior is most characteristic of the phenotypic region. Rigorous methods for obtaining interior points exist and are readily applicable to linear programming problems^9^. However, we have found a fairly simple strategy that has worked well. We (1) identify regions with parameters that are mathematically unbounded and we bound the corresponding parameters to biologically realistic values; (2) use a set of parameter values at a vertex of the feasible region as the starting values; (3) perform a line search using linear programming to find the boundary values for parameter p_i_; (4) update the starting values by the geometric mean between boundaries; (5) repeat the same procedure sequentially for each parameter. The entire sequence is then repeated in a daisy-chain fashion until the values converge, or the procedure terminates after a specified number of iterations.

We show an example of this method in Figure M2 for 6 qualitatively-distinct phenotype in a 2-D slice of design space. For each phenotypic region we show a sequence of steps, starting from a vertex, that results in a trajectory of interior points. The sequence of values tends to converge near the center of each phenotypic region. Although these phenotypic regions have simple geometries and thus the method converges within a few steps, this method can also be applied to identify interior points within phenotypic regions that are irregular and involve many dimensions. The end result is a set of parameter values capable of realizing any qualitatively-distinct phenotype of interest.

*Characteristics of qualitatively-distinct phenotypes*

There are many characteristics associated with any given phenotype, and these can be readily analyzed in terms of the S-system equations that define the qualitatively-distinct phenotypes. S-Systems are tractable by virtue of becoming linear when transformed to logarithmic coordinates^8^. There is a large body of work that deals with the analysis of S-Systems^11^. We can apply the power of S-Systems analysis to better understand the properties of the qualitatively-distinct phenotypes of the system. For example, the log-gain functions that characterize signal propagation, as well as parameter sensitivities that characterize the robustness of the system, are defined purely by architectural features (kinetic orders) of the S-System model and thus are constant throughout the entire region of validity for a given qualitatively-distinct phenotype, independent of parameter values. The log-gain of *X*_2_ in response to a change in *X*_3_ for each valid phenotype is shown in the penultimate column of Table M1. However, not all phenotypic characteristics are parameter independent. An example that is of particular interest in the realm of dynamical systems is the stability of the fixed points. This can be calculated automatically using the Routh criteria for stability^8^, which identify the number of eigenvalues with positive real part. The stability of the fixed points can be determined automatically and the results for our simplified model are shown in the last column of Table M1. These are only a small sample of the characteristics associated with any phenotype that can be examined in this fashion.

***Part 3: Identification of Parameter Sets for Co-Localized Phenotypes***

A critical issue in many studies is the transition between specific phenotypes as certain parameters change; in such cases it is important to have the phenotypes co-localized within a given slice of design space so that the transition can be easily visualized and characterized. However, the task of finding values for the remaining parameters that are fixed can be a challenge. For example, assume we have a model with three parameters, *x, k* and *z* and two phenotypes of interest, as shown in Figure M3a, where one phenotype might represent a normal physiological state (green) and the other an abnormal state (red). The *x* and *z* parameters might represent genotypically determined parameters, while the *k* parameter might correspond to the concentration of a specific environmental toxin. In this example, we are searching for a value of the *k* parameter such that changes in *x* and *z* (the slice parameters) produce a transition between the normal and abnormal phenotypes. Intuitively, this is equivalent to taking 2-dimensional slices through design space until finding those that contain both the green and the red phenotypes. An example of a 2-D slice in which both phenotypes are co-localized is shown in Figure M3a with *k*  = *k*_0_.

How might this solution be efficiently implemented? A naïve approach might involve sampling *n* points within specific ranges of the slice parameters *x* and *y*. This defines an *n^2^* mesh and the behavior of the model at each point within this mesh could be calculated. If the behaviors to be co-localized are not identified within this *n^2^* mesh, then a new value of the non-slice parameter *k* is chosen and the analysis repeated until the behaviors are co-localized, or until a stop criterion is reached after a large number of iterations. Consider what this entails when seeking to co-localize a set of phenotypes for a larger system with a total of 10 parameters of which 2 are allowed to change. Sampling just three values for each of the 8 non-slice parameters and a mesh of points for the 2 slice parameters would involve unique samples. This represents a huge sampling problem that minimally explores parameter space; furthermore, it does not guarantee that the behaviors are found co-localized within a slice. Clearly, this naïve approach of sampling parameter values is not adequate for the task.

*Simple Example*

By contrast, the co-localization of phenotypes in the system design space framework can be achieved without the need for sampling parameter values. We illustrate this part of the strategy first with a simple concrete example before providing a formal description that covers the general case. The intuitive approach described in the first paragraph of this section viewed the co-localizing of the two phenotypes in Figure M3a by means of a 2-D slice through the 3-D design space. Assume that the goal now is to illustrate the co-localization of normal and abnormal phenotypes in a 1-D slice involving only the parameter *x*. Each phenotype has a single boundary condition consisting of a plane that divides the 3-D space into two half-spaces (recall that qualitatively-distinct phenotypes have linear boundaries in logarithmic coordinates, e.g. Eqs. 21-25). The boundaries for the two phenotypes (green and red) are given in order by the following inequalities,

[27]

and

[28]

where *c*_1_ represents a positive constant. The value of the slice parameter must not be the same for the two phenotypes; thus, we define two auxiliary variables, *s*_1_ and *s*_2_, where *s*_1_ corresponds to for the green phenotype, and *s*_2_ corresponds to for the red phenotype*.* Next, substitute *s*_1_ and *s*_2_ into Eq. 27 and Eq. 28, respectively, and combine the result to form the boundaries with the auxiliary variables in matrix notation,

[29]

This system of inequalities has 4 variables and thus we have mapped the original boundaries in a lower-dimensional space into a higher-dimensional space. The system of linear inequalities in Eq. 29 defines a feasible region in this 4-D space. A single point within this 4-D space can be projected back onto the original 3-D space as two distinct points that share the values for *z* and *k*, but have different values for *x*. A visual representation of this is shown in Figure M3b. If we allow *s*_1_ and *s*_2_ to vary, we obtain a 2-D space in which the axes correspond to the *s*_1_ and *s*_2_ variables. Each of the boundary conditions defines the feasible region for one phenotype, shown as green or red in Figure M3b. If the intersection of these regions exists, as it does in this example, it corresponds to the feasible region for the combined boundaries within which both phenotypes are valid (shown by the yellow region in Figure M3b). Thus, the co-localization of phenotypes in the system design space framework is achieved without sampling parameter values.

The graphical solution in Figure M3b can readily be translated into the corresponding 1-D view in Figure M3c. A single point within the feasible region of Figure M3b, for example the extreme point in the upper-left corner given by = *c*_1_ – log *k*_0_, and = 3*c*_1_ – log *k*_0_, corresponds to two points in the original 3-D space. The green phenotype is valid when log *x* = *c*_1_ – log *k*_0_, log *k* = log *k*_0_ and log *z* = 0; and the red phenotype is valid when log *x* = 3*c*_1_ – log *k*_0_, log *k* = log *k*_0_ and log *z* = 0. The 1-dimensional slice in Figure M3c depicts the two values of the slice parameter log *x* that stem from the single value in Figure M3b. Although this is a simple example, the same principle can be applied to problems with a large number of slice parameters and a large number of phenotypes that have more complicated boundary conditions.

*General Strategy*

The following is a more formal description of the general strategy. We have previously shown that cases in system design space can intersect, that the regions of intersection can be enumerated by combining the boundaries of the individual cases, and that validity of the combined set can be readily determined^5^. The task of determining whether *n* phenotypes are co-localized within an *m-*dimensional slice involves finding the intersection of cases in a higher dimensional space. This is achieved by mapping the boundary conditions onto a higher-dimensional manifold. This higher-dimensional manifold and the corresponding combined boundaries are obtained through a series of steps.

We start with the boundary conditions for the *i*-th case that are linear inequalities in logarithmic coordinates^5^, as shown in Eq. 26. To find whether *n* cases are co-localized within an *m-*dimensional slice, we must find *n* points that together satisfy the boundary conditions for all the cases. All of these points share the values of the *p* – *m* non-slice parameters. Thus, a partitioning of Eq. 26 into non-slice parameters and slice parameters yields

[30]

where the π subscript corresponds to the non-slice parameters and the *σ* subscript corresponds to the slice parameters.

If a given case is valid, then there exists a point in the *m-*dimensional slice that also lies within the feasible region of the case. The corresponding points for each of several valid cases need not be the same; therefore, we define a set of *n* auxiliary variables corresponding to the slice parameters for each of the *n* cases in the set. In this way, we find multiple points in the *m*-dimensional slice that determine the validity of multiple cases simultaneously. The boundary conditions for a single case can be written as

[31]

where *y_i,σ_* is a column vector containing logarithms of *m* auxiliary variables corresponding to the slice parameters for the *i*-th case. The boundary conditions for all the cases can be combined and represented in matrix form as

[32]

where *W*, *H*, *s*, and *ζ* are defined by the following block matrices and vectors,

[33]

[34]

[35]

and

[36]

The total number of auxiliary variables, *s*, is the product *n*⋅*m* and the number of dimensions for the new space is *p + m*⋅(*n* – 1). The resulting boundaries define a region in this higher dimensional space, and the feasibility of co-localization can be determined by solving the following linear programming problem,

*minimize ε*

*subject to – Wy – Hs –* [1, 1, …, 1]*^T^ ε ≤ ζ*

A feasible region exists if the slack variable, ε, can be minimized to a negative value. If the boundaries define a feasible region, then sets of parameter values can be obtained automatically. Although the auxiliary variables in *s* have been redefined, each corresponds to a slice parameter in *y_σ_* for a specific case*.* The projection of the *s* variables back onto the *m*-dimensional slice results in *n* unique points.

*Maximizing the number of phenotypes in a slice of design space*

The ability to automatically identify parameters for phenotypes that are co-localized within an *m*-dimensional slice of design space allows us to ask some interesting questions. For example, what is the maximum number of *n* qualitatively-distinct phenotypes that can be co-localized in a 2-dimensional slice of design space? Our solution is to apply the method described in the previous subsection. We (a) select 2 parameters of interest, (b) define the set of 2*n* independent slice parameters*,* and (c) combine all the boundary conditions for the complete set of the *n* valid phenotypes. If the set of phenotypes does not have a feasible region, we can remove individual phenotypes iteratively until a feasible region is found. Recall that the simplified model described by Eqs. 3-6 has 9 parameters and a total of 9 qualitatively-distinct phenotypes. If we combine all the boundaries of the valid phenotypes, we obtain a system of linear constraints in a 9 + 2⋅(9 – 1) = 25-dimensional space. Using linear programming we determine that the boundary conditions on this higher dimensional manifold define a feasible region in design space, thus there is a 2-D slice that contains all the behaviors of the system. We then automatically obtain a set of parameter values within this slice, as shown in Figure M4.

***Part 4: Identification of Ensembles and Ordered Sequences of Desirable Phenotypes***

The synthesis of any new system, biological or otherwise, involves engineering a particular design to exhibit specific behaviors that occur under specific operating conditions. In complex nonlinear systems, these behaviors are not typically local behaviors, but involve global properties of the system with multiple distinct phenotypes. Thus, we say that desired behaviors are generated from ensembles of distinct phenotypes that are observed at different values of the input parameters. The task of designing a biological system that behaves according to the desired ensemble of phenotypes can be challenging, again due to the complex nonlinearity of biological mechanisms. Our strategy enables us to identify groups of qualitatively distinct phenotypes with desired behaviors that are co-localized within some slice of design space, and to obtain values for parameters associated with the slice. The slice may be bounded, such that all the parameters are within certain ranges relevant to their biology and/or the desired range of operation.

This subsection provides two examples in which ensembles of the qualitatively-distinct phenotypes are automatically generated by (a) selecting an ensemble of phenotypes that exhibit behaviors of interest, (b) determining whether they are co-localized in a 1-dimensional slice, and (c) finding a set of parameter values associated with the desired ensemble of phenotypes. In both cases, the input concentration *X*_3_ is the slice parameter. In addition, the second example below shows an ensemble of cases that is ordered to produce a specific progression of behavior.

*Connecting three regions of bistability*

In the first example, we determine whether there is a 1-dimensional slice in which all the unstable phenotypes of the system are co-localized. From Table M1 we identify three qualitatively distinct phenotypes that exhibit an unstable behavior. The instability they exhibit is exponential instability, where the analysis of the local dynamics reveals that there is one positive eigenvalue, and all other eigenvalues have negative real parts. This type of instability is purely a function of the kinetic orders, and hence there is at least one positive eigenvalue for the entire region of validity, independent of parameter values. We select Cases 9, 13 and 14 and determine whether they are co-localized in the 1-D slice with *X*_3_ as the slice parameter. The result of our co-localization for the desired ensemble of phenotypes is shown in Figure M5a. The unstable phenotypes are always overlapped by stable phenotypes, which results in bi-stability for the entire slice.

*Designed induction characteristic with ordered phenotypes*

In the second example we identify a slice of design space where Cases 1, 5, 13, 15 and 16 are co-localized and arranged to produce a specific induction characteristic. These cases were chosen based on their log-gain of *X*_2_ with respect to *X*_3_, as shown in Table M1. The phenotypes have log-gains that correspond to (a) saturation of *X*_2_ (log-gain of 0), (b) synthesis of *X*_2_ within the regulatable domain (log-gain of 2), and (c) negative log-gain consistent with an unstable phenotype (as there are no negative feedback loops within the system) for a switch-like behavior. However, co-localization of these cases, as previously described, does not guaranteed a specific arrangement; thus, the resulting slice could exhibit any one of many potential induction curves, depending on the order in which the phenotypes become dominant as the concentration of *X*_3_ increases. To obtain an ensemble of cases that produces a specific progression of behavior as the slice variable, in this case *X*_3_, increases we add additional constraints among the auxiliary variables, *s*, during co-localization. By adding constraints to the combined set of boundaries, defined by Eq. 32, a particular arrangement can be specified and a set of parameters that satisfies the constraints automatically determined.

For example, If we now apply our method to identify sets of parameters where the phenotypes are co-localized, we obtain an array of new auxiliary variables *s* = {*s*_1_, *s*_2_, *s*_3_, *s*_4_, *s*_5_}, where *s*_1_ corresponds to log *X*_3_ for Case 1, and *s*_2_ corresponds to log *X*_3_ for Case 5, etc. We can identify a slice with a specific progression of behaviors by stating that certain slice variables in *s* are larger than others. For example, let us assume our intended design requires Case 1 to occur at very low concentrations of *X*_3_. As we increase *X*_3_ we require the system to become regulatable until switching to a higher state. Thus, *s*_2_, the *s* slice variable that corresponds to log *X*_3_ for Case 5, would be greater than *s*_1_. The following constraints are added to the combined set of boundary conditions to ensure that the correct progression of phenotypes is identified,

[37]

[38]

[39]

[40]

By adding the constraints in Eqs. 37-40 we identify a slice with the following progression of behaviors as *X*_3_ increases: basal rate of expression, followed by activation within the regulatable regime, followed by an unstable regime, followed by a second regulatable regime, and ultimately saturation. Because Case 13 is unstable, as shown in the last column of Table M1, we expect it to overlap with some of the other cases and generate instances of bi-stability. The progression of behaviors specified by Eqs. 37-40 is consistent with an overlap of Cases 5, 13 and 15. The result of our co-localization for the ordered ensemble of phenotypes to realize this induction characteristic is shown in Figure M5b.

This simple example can be extended for the ordering of cases in higher-dimensions by adding constraints among auxiliary variables for multiple slice parameters. Furthermore, the constraints may involve more complex relationships, as long as both sides of each constraint inequality involve a single positive product of power laws.

**AUTOMATIC IDENTIFICATION AND CHARACTERIZATION OF THE PHENOTYPIC REPERTOIRE FOR A CLASS OF GENETIC OSCILLATOR DESIGNS**

***Two-Gene Relaxation Oscillator***

The first test of our automated parameter-independent strategy involves the analysis of a relaxation oscillator design that had previously been analyzed in the conventional manner by first assembling experimentally measured and estimated parameter values. We analyzed the relaxation oscillator design following the global-to-local approach outlined in ^12^ and obtained the results shown in Figure 2 of the main text. The mechanistic model and the meaning of the parameters are reproduced here for convenience,

[41]

[42]

[43]

[44]

where *X*_1_ and *X*_2_ represent the concentration of activator mRNA and protein, respectively; *X*_3_ and *X*_4_ represent the concentration of repressor mRNA and protein, respectively. The *α* and *β* parameters are rate constants for synthesis and degradation; the *ρ* parameters represent the capacity for regulation; the *K*_2R_ and *K*_2_ parameters are the concentrations of activator for half-maximal induction of repressor and activator transcription; the *K*_4A_ parameter is the concentration of repressor for half-maximal repression of activator transcription. The kinetic orders associated with activator and repressor binding are assumed to be 2, consistent with two sites for regulator binding. The architecture of the network is conserved for *ρ*_1_ > 1 and *ρ*_3_ > 1.

We used the strategy outlined in the main text and expanded upon in the previous section to analyzed the design^13^ represented by Eqs. 41-44. Following the specification of the system architecture above, the second step in the strategy is the recasting of the original equations, which involve rational function nonlinearities, to generate equations in the generic GMA form. This involves defining new auxiliary variables for the terms in the denominators of Eqs. 41 and 43, and expanding the terms in the numerators. The result is the following set of differential equations plus algebraic constraints,

[45]

[46]

[47]

[48]

[49]

[50]

We applied the system design space methodology to achieve the phenotypic deconstruction of the system, which yields a maximum of 36 potentially valid qualitatively-distinct phenotypes. We determined validity using linear programming^5^ and found a total of 15 (valid) qualitatively-distinct phenotypes. In step 3, sets of parameter values for each of these qualitatively-distinct phenotypes were determined automatically. The resulting phenotypes with their case number, case signature, and number of eigenvalues with positive real part are shown in Table 1 of the main text. The number of eigenvalues with positive real part was determined using the Routh criteria for stability^8^ applied within the system design space framework^7^.

Our analysis of the global repertoire of potential behaviors revealed a single phenotype that had the potential to oscillate. We determined a set of parameter values that is within the region of validity for this phenotype, and analyzed the phenotypic landscape around this point (Figure 2a of the main text). The automatically determined values for the parameters are: *α*_1_ = 3.162, *α*_2_ = 1, *α*_3_ = 1, *α*_4_ = 1, *β*_1_ = 1, *β*_2_ = 1, *β*_3_ = 1, *β*_4_ = 1, *ρ*_1_ = 100, *ρ*_3_ = 100, *K*_2_ = 3.162×10^–1^, *K*_2R_ = 1 and *K*_4A_ = 3.126×10^–2^.

Local analysis of the qualitatively-distinct phenotypes showed a region of potential oscillation, sandwiched between regions of overlapping stable and exponentially unstable phenotypes (Figure 2b of the main text). We focused our analysis of the full model within the region of potential oscillation and found that the system indeed oscillates (Figure 2c of the main text). Through our automated strategy, we have reproduced previous findings regarding this oscillator design, including its capacity to exhibit phenotypes that are stable, exponentially unstable, and oscillatory^7,12,13^.

***General Class of Two-Gene Oscillator Designs***

We have previously defined a general class of two-gene circuits involving an activator and a repressor^12^. This general class of circuits was previously used to compare 7 unique oscillator designs with close analogs that have been synthesized experimentally [e.g. ^13–16^]. However, there are 9 additional designs within this class that our previous analysis did not find to oscillate under the conditions used to ensure a fair comparison among the alternative designs. The mathematical models are very similar to that in Eqs. 41-44, but are modified to include an additional step in the maturation of the proteins^7^.

Here we revisit this general class of circuit designs, which are represented by the following mechanistic model that we reproduce here for convenience,

[51]

[52]

[53]

[54]

[55]

[56]

where *X*_1_, *X*_A_ and *X*_2_ represent the concentration of activator mRNA, immature protein and functional protein, respectfully; *X*_3_, *X*_R_ and *X*_4_ represent the concentration of repressor mRNA, immature protein and functional protein, respectfully. The *α* and *β* parameters are the first-order rate constants for synthesis and degradation; µ is the exponential growth rate of the cells; the *ρ* parameters represent the capacity for regulation; the *K*_2R_ and *K*_2_ parameters are the concentrations of activator for half-maximal induction of repressor and activator transcription; the *K*_4_ and *K*_4A_ parameters are the concentrations of repressor for half-maximal repression of repressor and activator transcription. The *π* and *δ* are binary indices that can assume either a 0 or 1 value that define the mode of transcriptional control. The *π* parameters determine whether the primary mode of transcriptional control involves an activator (*π* = 1) or a repressor (*π* = 0). The *δ* parameters determine whether transcriptional control involves dual (*δ* = 1) or single (*δ* = 0) regulators. However, this mathematical model has been defined such that activator-only control of activator and repressor-only control of repressor are not allowed; instead, the combination *δ*_1_ = 0 and *π*_1_ = 1 (or *δ*_3_ = 0 and *π*_3_ = 0) indicates a constitutive mode of transcription control for the activator (or repressor). Each combination of values for *π*_1_, *δ*_1_, *π*_3_, and *δ*_3_ defines a design within this general class and the total number of combinations yields 16 distinct designs. For example, the design for the relaxation oscillator in the previous section, but with the added steps for protein maturation, is represented by *π*_1_ = 1, *δ*_1_ = 1, *π*_3_ = 1, and *δ*_3_ = 0.

In our previous analysis, we explored the oscillatory potential of seven of the designs in this general class by carefully determining nominal parameter sets from estimates and experimental measurements. These parameter sets were based on a rigorous procedure that involved (a) identifying experimental values available from the literature, (b) normalizing a reference system such that the cooperativity was at its greatest, thus maximizing the potential for oscillation, and (c) controlling for differences amongst the alternative designs^12^. The model for this general class, normalized and recast, is given by the following set of differential equations plus constraints,

[57]

[58]

[59]

[60]

[61]

[62]

[63]

[64]

where the superscript (^0^) corresponds to the normalized values for the parameters. The oscillatory phenotypes were then studied by varying the rate constants for effective degradation of activator and repressor proteins, *β*_2_ and *β*_4_, and for maturation of activator and repressor, *β*_A_ and *β*_R_. The *β*_2_ and *β*_4_ parameters mimic the effect of gratuitous inducers, which are used to tuned the behavior of synthetic biology constructs by altering the rate of formation of inactive complexes [e.g. ^15^]. The *β*_A_ and *β*_R_ parameters were varied to determine the effect of relative delays in activator and repressor maturation. Taken together, these four (free) parameters were used to explore the characteristics of the oscillatory phenotypes. In order for these designs to remain tunable and biologically relevant, the systems were analyzed when *β* > *μ* for all *β* = {*β*_1_, *β*_A_, *β*_2_, *β*_3_, *β*_R_, *β*_4_}. With these constraints, the second negative term in each equation can never be dominant. Hence, we can effectively remove these terms from the model for the design space analysis (but not for simulating the dynamics of the full system).

Here, we use the same equations and the same strategy for consistency, but in contrast to our previous analysis, the parameters of the system have not been estimated or experimentally measured. Instead, sets of parameter values are determined completely automatically using the design space framework. Once a set of parameter values has been automatically determined, we vary the four free parameters in a 4-D lattice around this point in design space and apply the Routh criteria for stability to identify the phenotypes that have potential for oscillatory behavior.

Thus, each design was analyzed in an automated manner by (a) enumerating the qualitatively-distinct phenotypes, (b) determining a set of parameter values within the valid region of each phenotype, and (c) determining the potential for oscillation. The results of our analysis for the 16 designs are summarized in Table 2 of the main text.

**REFERENCES**

1. Arkin, A., Shen, P. & Ross, J. A Test Case of Correlation Metric Construction of a Reaction Pathway from Measurements. *Science* **277,** 1275–1279 (1997).

2. Ross, J. From the determination of complex reaction mechanisms to systems biology. *Annu. Rev. Biochem.* **77,** 479–494 (2008).

3. Hlavacek, W. S. & Savageau, M. A. Subunit structure of regulator proteins influences the design of gene circuitry: analysis of perfectly coupled and completely uncoupled circuits. *J. Mol. Biol.* **248,** 739–755 (1995).

4. Savageau, M. A. & Voit, E. O. Recasting nonlinear differential equations as S-systems: a canonical nonlinear form. *Math. Biosci.* **87,** 83–115 (1987).

5. Fasani, R. A. & Savageau, M. A. Automated construction and analysis of the design space for biochemical systems. *Bioinformatics* **26,** 2601–2609 (2010).

6. Savageau, M. A., Coelho, P. M. B. M., Fasani, R. A., Tolla, D. A. & Salvador, A. Phenotypes and tolerances in the design space of biochemical systems. *Proc. Natl. Acad. Sci. U. S. A.* **106,** 6435–6440 (2009).

7. Lomnitz, J. G. & Savageau, M. A. Phenotypic deconstruction of gene circuitry. *Chaos* **23,** 025108 (2013).

8. Savageau, M. A. *Biochemical Systems Analysis: A Study of Function and Design in Molecular Biology, 40th Anniversary Edition*. (Createspace, 2009) [a reprinting of the original edition (Addison-Wesley Publishing, 1976)].

9. Vanderbei, R. J. *Linear Programming: Foundations and Extensions*. (Springer, 1996).

10. Dantzig, G. B. *Linear Programming and Extensions*. (Princeton University Press, 1965).

11. Voit, E. O. Biochemical Systems Theory: A Review. *Int. Sch. Res. Not.* **2013,** e897658 (2013).

12. Lomnitz, J. G. & Savageau, M. A. Strategy Revealing Phenotypic Differences among Synthetic Oscillator Designs. *ACS Synth. Biol.* **3,** 686–701 (2014).

13. Atkinson, M. R., Savageau, M. A., Myers, J. T. & Ninfa, A. J. Development of genetic circuitry exhibiting toggle switch or oscillatory behavior in Escherichia coli. *Cell* **113,** 597–607 (2003).

14. Elowitz, M. B. & Leibler, S. A synthetic oscillatory network of transcriptional regulators. *Nature* **403,** 335–338 (2000).

15. Stricker, J. *et al.* A fast, robust and tunable synthetic gene oscillator. *Nature* **456,** 516–519 (2008).

16. Tigges, M., Marquez-Lago, T. T., Stelling, J. & Fussenegger, M. A tunable synthetic mammalian oscillator. *Nature* **457,** 309–312 (2009).

Table M1. The Phenotypic repertoire with characteristics for the model in Figure M1.

| **Case #** | **Case Signature** | **Validity** | **∂log *X*_2_/**  **∂log *X*_3_** | **Stability** |
| --- | --- | --- | --- | --- |
| 1 | 11111111 | + | 0 | S |
| 2 | 11111121 | – | – | – |
| 3 | 11112111 | – | – | – |
| 4 | 11112121 | – | – | – |
| 5 | 11211111 | + | 2 | S |
| 6 | 11211121 | + | 0 | S |
| 7 | 11212111 | – | – | – |
| 8 | 11212121 | – | – | – |
| 9 | 21111111 | + | 0 | U |
| 10 | 21111121 | – | – | – |
| 11 | 21112111 | + | 0 | S |
| 12 | 21112121 | – | – | – |
| 13 | 21211111 | + | -2 | U |
| 14 | 21211121 | + | 0 | U |
| 15 | 21212111 | + | 2 | S |
| 16 | 21212121 | + | 0 | S |

The complete set of potential qualitatively-distinct phenotypes is given with their case numbers and signatures. Nine are valid and correspond to the repertoire of qualitatively-distinct phenotypes. Two characteristics of each phenotype are shown: logarithmic gain (; 2, 0, -2) and stability (stable, S; unstable, U). See text for discussion.

**Figure M1. Schematic diagram for a toy model of a cascade of two activators and within a positive feedback loop subject to modulation by an environmental variable .** Horizontal arrows represent mass flow, vertical arrows represents activation of target processes.

**Figure M2. Example of the method for obtaining interior parameter sets for each of six phenotypic regions in a two dimensional slice of design space.** The independent variable, *X*_3_, is plotted on the x-axis, and the concentration of activator for half-maximal induction, *K*_2_, of *X*_1_ synthesis is plotted on the y-axis. The colored regions represent qualitatively-distinct phenotypes. Color bar identifies the case numbers. The axes are in logarithmic coordinates and are normalized about a value automatically determined for the phenotype represented by Case 5. A trajectory of interior points (white dots) obtained by a sequence of line searches in orthogonal directions (black lines), starting from initial values at a vertex (white square), until it converges in the center of a region (white star).

**Figure M3. Example of the strategy for obtaining sets of parameter values that result in co-localization of phenotypes.** (a) The feasible regions of two phenotypes, represented by the green and red polyhedrals, in a 3-D design space. Three examples of 2-D slices are shown for different values of the *k* parameter. (b) The mapping of the lower-dimensional boundaries onto a higher dimension showing the auxiliary variables *s*_1_ and *s*_2_ that correspond to log *x* for the green and red phenotypes, respectively. The green and red areas represent values of the auxiliary variables where each phenotype is valid. The overlapping region in the lower-right hand corner represents a feasible region for both phenotypes. The slice within which the phenotypes are co-localized is found automatically. (c) The 1-D slice with log *k* = log *k*_0_ and log *z* = 0. The single point shown in panel B is mapped back to the original space of the *x* parameter where the point becomes two points, shown by the white and black circles.

**Figure M4. Example of a 2-D slice of design space that contains the complete phenotypic repertoire of the system in Figure M1.** The axes are normalized with respect to the values automatically determined for Case 13 (white square). The values of the non-slice parameters are *α*_1_ = 3.162×10^–3^, *α*_2_ = 1, *ρ*_1_ = 100, *ρ*_2_ = 100, *β*_1_ = 1, *β*_2_ = 1 and *X*_3_ = 3.162×10^–1^.

**Figure M5. Examples of specific ensembles of behaviors constructed automatically.** The x-axis represents the 1-D slice parameter, the concentration of *X*_3_, in log coordinates normalized with respect to the values automatically determined for Case 13. The y-axis represents the phenotypic character, the logarithm of the steady-state value for the activator *X*_2_. Solid lines represent stable fixed points; dashed lines represent unstable fixed points. The top bar shows the case numbers and the colors correspond to those in Figure M4. (a) All the unstable phenotypes co-localized in a 1-D slice. (b) A specific induction characteristic exhibiting hysteresis determined by ordering Cases 1, 5, 13, 15 and 16. Note that the unstable phenotypes 9, 13 and 14 only appear in regions of overlap.
